# NO-sGC Pathway Modulates Ca^2+^ Release and Muscle Contraction in Zebrafish Skeletal Muscle

**DOI:** 10.3389/fphys.2017.00607

**Published:** 2017-08-23

**Authors:** Zhou Xiyuan, Rainer H. A. Fink, Matias Mosqueira

**Affiliations:** ^1^Medical Biophysics Unit, Institute of Physiology and Pathophysiology, Heidelberg University Hospital Heidelberg, Germany; ^2^Department of Traumatic Surgery, TongJi Hospital affiliated to TongJi Medical College, Huazhong University of Science and Technology Wuhan, China

**Keywords:** skeletal muscle, force, calcium transient, nitric oxide, zebrafish

## Abstract

Vertebrate skeletal muscle contraction and relaxation is a complex process that depends on Ca^2+^ ions to promote the interaction of actin and myosin. This process can be modulated by nitric oxide (NO), a gas molecule synthesized endogenously by (nitric oxide synthase) NOS isoforms. At nanomolar concentrations NO activates soluble guanylate cyclase (sGC), which in turn activates protein kinase G via conversion of GTP into cyclic GMP. Alternatively, NO post-translationally modifies proteins via S-nitrosylation of the thiol group of cysteine. However, the mechanisms of action of NO on Ca^2+^ homeostasis during muscle contraction are not fully understood and we hypothesize that NO exerts its effects on Ca^2+^ homeostasis in skeletal muscles mainly through negative modulation of Ca^2+^ release and Ca^2+^ uptake via the NO-sGC-PKG pathway. To address this, we used 5–7 days-post fecundation-larvae of zebrafish, a well-established animal model for physiological and pathophysiological muscle activity. We evaluated the response of muscle contraction and Ca^2+^ transients in presence of SNAP, a NO-donor, or L-NAME, an unspecific NOS blocker in combination with specific blockers of key proteins of Ca^2+^ homeostasis. We also evaluate the expression of NOS in combination with dihydropteridine receptor, ryanodine receptor and sarco/endoplasmic reticulum Ca^2+^ ATPase. We concluded that endogenous NO reduced force production through negative modulation of Ca^2+^ transients via the NO-sGC pathway. This effect could be reversed using an unspecific NOS blocker or sGC blocker.

## Introduction

In skeletal muscle, Ca^2+^ release from the sarcoplasmic reticulum (SR) is triggered when dihydropyridine sensitive voltage dependent L-type Ca^2+^ channels (DHPR) mechanically activate the ryanodine receptor (RyR) type 1 (Stephenson et al., 1998). Alternatively, DHPR-mediated Ca^2+^ current also induces Ca^2+^ release via activation of RyR type 3 (Ca^2+^ induced Ca^2+^ release, CICR; Koulen et al., 2001). The Ca^2+^ release from the SR via the RyR increases the free cytosolic Ca^2+^ concentration ([Ca^2+^]_i_) about 100-fold enabling the shortening of the myocyte myofilaments (Stephenson et al., 1998). After Ca^2+^ release, the contraction process is terminated as a result of Ca^2+^ uptake mechanisms, removing Ca^2+^ from the cytosol primarily back into the SR mediated by SR Calcium ATPase (SERCA) and secondarily extruded into the extracellular space by a sodium-calcium exchanger (NCX). This dynamic and fast Ca^2+^ movement from the SR into the myoplasm and then back into the SR is denominated Ca^2+^ transient. The time course and the amplitude of the Ca^2+^ transients determine force production (Stephenson et al., 1998; Baylor and Hollingworth, 2012; Hollingworth et al., 2012).

Although, both force production and Ca^2+^ homeostasis processes are well-described, several modulators have been identified, but their role on muscle contraction are not completely understood. Nitric Oxide (NO) is a small bioactive gas molecule, with complex arrays of message intermediation *in vivo* (Ignarro et al., 1987) that actively modulates force production and Ca^2+^ homeostasis. Its effect is dose dependent, where high concentrations induce an increase of force production via increase of Ca^2+^ release, whereas low concentrations induce a reduction of force. NO is synthesized endogenously from the catalytic action of NO synthase (NOS; Knowles and Moncada, 1994; Nathan and Xie, 1994), whereas a small fraction of NO can be released from nitrovasodilators such as glyceryl-trinitrate (nitroglycerin) or the redox reaction of nitrite under acidic conditions (Nakahira et al., 2010). So far four NOS isoforms have been described in mammalian cells: neuronal NOS (nNOS or NOS1) and endothelial NOS (eNOS or NOS3), are both constitutively expressed and activated by Ca^2+^/calmodulin; inducible NOS (iNOS or NOS2), whose activity is Ca^2+^ independent (Knowles and Moncada, 1994; Nathan and Xie, 1994) and mitochondrial NOS (mtNOS), as α-isoform of nNOS, is constitutively expressed at the inner membrane of mitochondria and is considered the fourth isoform (Giulivi, 1998; Giulivi et al., 1998; Tatoyan and Giulivi, 1998; Carreras et al., 2007). In skeletal muscle fibers, nNOS is located in cytoskeletal structures or plasma membranes through binding to the dystrophin-associated protein, α1-syntrophin (Wakayama et al., 1997) and is highly concentrated at the inner surface of the sarcolemma, subsarcolemmal areas near neuromuscular junctions, myotendinous junctions and costamers (Chang et al., 1996; Grozdanovic and Gossrau, 1998). Zebrafish express two types of NOS: NOS1 and NOS2, which are highly conserved in evolution, showing similar amino acid sequences, cofactor binding domains, specific domains for each isoform and intron position and phases (Andreakis et al., 2011). Although, both larvae and adult zebrafish express NOS1 in the brain and peripheral nervous system (Holmqvist et al., 2000, 2004; Shin et al., 2000; Poon et al., 2003), where NO/cGMP can modulate formation and maturation of neuromuscular junctions (Jay et al., 2014), this isoform has not yet been characterized in skeletal muscle of the zebrafish. NOS2 is widely expressed in all zebrafish tissues in two different isoforms: NOS2a, which is expressed after LPS injection in adult zebrafish and NOS2b, which presents a N-terminal myristoylation sequence and is constitutively expressed in all tested tissues, suggesting that NOS2a behaves as a classical iNOS and NOS2b is similar to the mammalian eNOS (Lepiller et al., 2009).

To date, the impact of NO on the modulation of muscle systems is still under evaluation. At nanomolar levels NO activates soluble guanylyl cyclase (sGC), increasing cGMP levels, which leads to activation of a myriad of physiological processes such the activation of PKG, cGMP-cation gated channels, cGMP-hydrolyzing phosphodiesterases (PDE), and cGMP-hydrolyzing PDEs (Francis et al., 2010). NO-sGC-cGMP negatively modulates contractility, accelerates relaxation, and improves the stiffness of myocytes via direct PKG phosphorylation of proteins, including troponin I, L-type Ca^2+^ channel, phospholambam, titin, inositol triphosphate receptor, Na^+^/K^+^-APTase α-subunit, PDE5, ryanodine receptor, transient receptor potential Ca^2+^ channel isoform 3 and 6, among others (Francis et al., 2010). The second pathway is denominated S-nitrosylation (SNO), where NO interacts with free reactive cystein thiol groups to form S-nitrosothiols (Anzai et al., 2000; Eu et al., 2000; Stamler and Meissner, 2001; Sun et al., 2001). Although, zebrafish have been used as an animal model for skeletal muscle patho-physiological approaches, the precise mechanisms of NO action on the zebrafish Ca^2+^ homeostasis during muscle contraction are still unclear. Based on previous works, we hypothesized that in skeletal muscle of zebrafish larvae NO exerts its effects on Ca^2+^ homeostasis during muscle contraction mainly through negative modulation of Ca^2+^ release and Ca^2+^ uptake via the NO-sGC-PKG pathway. To address this, we used 5–7 days-post fecundation-larvae of zebrafish, a well-established animal model to study formation, development, and motor innervation as well as to comprehend molecular and cellular aspect of several myopathies (Schredelseker et al., 2010; Muto et al., 2011; Jay et al., 2014). In our study, we observed that nNOS is expressed in skeletal muscle and the Ca^2+^ transients recorded were limited to the sarcolemmal. Exogenous NO donors reduced force production via negative modulation of Ca^2+^ transients via the NO-sGC-PKG pathway, while blocking endogenous NO synthesis or the action of sGC produced the opposite effect.

## Materials and methods

### Animals

Wild type zebrafish larvae, 5–7 days post fecundation (dpf), were used. All animal experiments have been performed in accordance with the guidelines of the state of Baden–Wuerttemberg and it has also been approved by the ethics committee of the University of Heidelberg Interfaculty Biomedical Research Facility (T54/16).

### Force measurements in zebrafish larvae

The force measurement protocol using zebrafish larvae was described in detail elsewhere (Dou et al., 2008). Briefly, 5–7 dpf zebrafish larvae were anesthetized using 4°C modified Krebs–Henseleit (MKH, in mM: 117.2 NaCl, 25.2 NaHCO_3_, 4.7 KCl, 1.2 MgCl_2_, 1.2 KH_2_PO_4_, 2.5 CaCl_2_, and 11.1 Glucose, pH 7.4) solution. The head was then removed with microsurgical scissors and forceps under the microscope (Olympus, SZ60). Two pieces of aluminum foil (both 2 ^*^ 8 mm) with cyanoacrylate glue were prepared and bent to a right angle. Anterior and caudal sections were set on the aluminum foil and mounted into the force transducer immersed in freshly prepared MKH and bubbled with 5% CO_2_/95% at room temperature. After fixation to the force transducer, the preparation was incubated in a glass chamber, which was perfused continuously (4 ml/min) with MKH. Two platinum electrodes flanking the preparation provided electrical field stimulations controlled by an isolated pulse stimulator (A-M System, model 2100) and a Grass SD9 stimulation unit. The mechanical signals from the force transducers were digitally converted by a PowerLab 15T AD converter (AD Instruments) and sampled (1 kHz sampling frequency) with LabChart 7 software (AD Instruments) and stored in a computer. The optimal length was established adjusting the length between the force transducer and the holder applying supramaximal stimulation in order to establish rheobase and chronobase for each experiment. Force-frequency curves were recorded with 200 ms stimulation trains at 2 ms pulse duration and supramaximal voltage, in 2 min interval. The stimulation frequency was increased in steps (1, 5, 10, 15, 30, 45, 60, 90, 120, 150, 180, and 200 Hz).

### Pharmacological agents

All pharmacological agents were purchased from Sigma-Aldrich if not stated otherwise. NO donor S-Nitroso-N-acetyl-DL- penicillamine (SNAP) 100 μM and Diethylamine NONOate diethylammonium salt (DEA-NONOate; D5431) 1 μM, unspecific NO blocker N^ω^-Nitro-L-Arginine Methyl Ester Hydrochloride (L-NAME) 5 mM, caffeine (250 μM), verapamil hydrochloride (20 μM), cyclopiazonic acid (CPA) 2.5 μM, NiCl_2_ 2 mM, 1H-[1,2, 4]oxadiazolo[4,3,-a]quinoxalin-1-one (ODQ) 10 μM, and N-ethylmaleimide (NEM) 100 μM.

### Isolation of skeletal myocytes from zebrafish larvae

Isolated skeletal muscle cells were obtained by adapting the procedure developed by Liao and Jain (2007). A pool of 20 zebrafish larvae were anesthetized and sacrificed as described above and skeletal muscles were incubated for 45 min at room temperature (25 ± 1.5°C) in digestion solution (in mM: 100 NaCl, 10 KCl, 1.2 KH_2_PO_4_, 4 MgSO_4_, 50 Taurin, 20 Glucose, 10 HEPES with 0.75 mg/ml Collagenase Type II; 4 mg/ml BSA, pH 6.9). A mix of transfer buffer A (TBA) and transfer buffer B (TBB) was used to slowly increase Ca^2+^ concentration. TBA (in mM): 117.2 NaCl, 25.2 NaHCO_3_, 4.7 KCl, 1.2 MgCl_2_, 1.2 KH_2_PO_4_, 11.1 Glucose, 10 HEPES, 10 BDM, and 5 BSA, pH 7.4. As control solution, TBB (in mM) was used: 117.2 NaCl, 25.2 NaHCO_3_, 4.7 KCl, 1.2 MgCl_2_, 1.2 KH_2_PO_4_, 2.5 CaCl_2_, 11.1 Glucose, 10 HEPES, and 5 BSA, pH7.4. The cell suspension was then centrifuged for 2 min at 500 rpm and the cell pellet was re-suspended in transfer buffer (TB)-1 (4.9 ml TBA in 0.1 ml TBB). This procedure was twice repeated in TB-2 (4.5 ml TBA in 0.5 ml TBB) and in TB-3 (2.5 ml TBA in 2.5 ml TBB). The cells were then seeded on 22 mm coverslip covered with 50 μl ECM placed on a 35 mm petri dish and incubated at 5% CO_2_ at 25 ± 1.5°C for 30 min prior use.

### Calcium transients in isolated skeletal myocytes

Isolated myocytes were incubated for 30 min with Fluo-4 AM (Molecular Probes, catalog number F14201) diluted to a final concentration of 10 μM in TBB. After incubation, the cells were washed once with TBB and the imaging dishes were mounted to a fluorescence microscope (Olympus Second Generation OSP-3 system) equipped with a photomultiplier unit and a Xenon light source system, filtered to provide an excitation wavelength of 488 nm. A photomultiplier was used to have a continuous recording of the fluorescence signal and thus a better time resolution necessary for the analyses of the biophysical parameters. An area of 7.5 μm^2^ was selected to record the Ca^2+^ transients from each cell. In order to introduce myocyte stimulation, a pair of platinum electrodes connected to a stimulator was placed to flank the cells. Three consecutive stimulations at 0.5 Hz 18 V for 10 ms were given and the change in fluorescence intensity was AD-converted (Powerlab 15T, AD Instruments) and sampled at 10 KHz in a PC using LabChart Pro7 software (AD Instruments). The analyses of calibrated Ca^2+^-transients were done using the Peak Analysis package from LabChart Pro 7. The traces were analyzed using 8 biophysical parameters: Peak (from baseline to maximum point of the trace: for force measurement in μN; for Ca^2+^ transients measurement in nM), Duration (total time of the force or Ca^2+^ transient, ms), Duration_50_ (duration of the force or Ca^2+^ transient at 50% of the peak, ms), Peak Area (area under the curve: for force measurement in μN^*^s; for Ca^2+^ transients measurements in nM^*^ms), Time to Peak (time from the beginning of the force or Ca^2+^ transient until the maximum, ms), Slope (rate of signal increase during time to peak: for force measured between 1 and 5 ms, μN/s; for Ca^2+^ transient measured between 0.5 and 2.5 ms, nM/s), Relaxation Time in force or Uptake Time for Ca^2+^ transients (time form maximum back to baseline, ms) and Tau (exponential decay from 90 to 10% of the response, ms). At the end of each experiment, the same cells were used to convert Fluo-4 fluorescence intensity into [Ca^2+^]_i_, as described previously (Grynkiewicz et al., 1985; Williams and Allen, 2007). Briefly, the cells were incubated with a high Ca^2+^-concentration solution that contained (in mM) 140 LiCl, 5 KCl, 1.2 KH_2_PO_4_, 1.2 MgCl_2_, 4 CaCl_2_, 20 HEPES, 0 EGTA, 0.005 Ionomycin, 0.01 CPA, 5 Caffeine and 1 Oubain and the resulting fluorescence was designated F_max_. Cells were then exposed to a similar solution, but now containing a Ca^2+^-free solution that contained (in mM) 140 LiCl, 5 KCl, 1.2 KH_2_PO_4_, 1.2 MgCl_2_, 0 CaCl_2_, 20 HEPES, 4 EGTA, 0.005 Ionomycin, 0.01 CPA, 5 Caffeine and 1 Oubain and the resulting fluorescence was designated F_min_. The following equation was used to convert Fluo-4 fluorescence readings F into [Ca^2+^]_i_:

$$\begin{array}{l} {\left[Ca^{2+}\right]}_{i}={\text{K}}_{\text{d}}\;\left(\text{F}-{\text{F}}_{\text{min}}\right)/\left({\text{F}}_{\text{max}}-\text{F}\right) \end{array}$$

The Fluo-4 dissociation constant (K_d_) of 345 nM was assumed according to the manufacturer's declaration.

### Line-scan of Ca^2+^ transient in isolated skeletal myocytes using a confocal laser scanning microscope

Isolated myocytes loaded with Fluo-4 as described previously were mounted onto a confocal laser scanning microscope (Olympus, Fluoview 300 System) perfused with TBB. Field electrical stimulation was performed as described above (15 V, 10 ms duration). The line scan trace was set at 1,024 pixels every 0.2 ms at the center of the myocyte's short axis and was moved down from 0 to −5 μm, in 1 μm steps in vertical direction. Continuous line scan was performed to monitor the change of the fluorescence intensity (according to the deepness of the confocal line-scan). The signal was recorded and processed using FLUOVIEW (Version 3.3.16) software.

### Immunofluorescence of isolated skeletal myocytes from zebrafish larvae

Isolated myocytes seeded on poly-L-lysine coated coverslips were fixed with ice-cold methanol for 5 min and rinsed twice with 1x PBS (in mM: 137 NaCl, 2.7 KCl, 10 Na_2_HPO_4_, and 2 KH_2_PO_4_, adjust pH to 7.4), permeabilized with 0.25% Triton X-100 in PBS and rinsed three times with 1x PBS. To block unspecific binding sites, myocytes were incubated for 30 min with 1x PBS containing 0.01% Tween 20 and 1% BSA. Diluted first anti-bodies were incubated over night at 4°C, rinsed with 1x PBS three times for 5 min following the incubation of the secondary antibody for 1 h at room temperature and finally rinsed with 1x PBS three times for 5 min. The samples were mounted with Dako mounting media according to the manufacturer's instructions and analyzed in a fluorescence microscope. The immunofluorescence pictures were taken at the sub-sarcolemmal region of the isolated myocyte. The primary and secondary antibodies were purchased from Abcam and diluted as follows: anti-CACNA1S antibody [1A], cat no. ab2862, dilution 1: 100; anti-Ryanodine Receptor antibody [C3-33], cat no. ab2827, dilution 1: 200; anti-SERCA1 ATPase antibody [VE121G9], cat no. ab2819, dilution 1: 200; anti-nNOS (neuronal) antibody, cat no. ab5586, dilution 1: 200; Donkey Anti-Mouse IgG H&L (Alexa Fluor® 647), cat no. ab150107, dilution 1: 500; Goat Anti-Rabbit IgG H&L (Alexa Fluor® 488), cat no. ab150077, dilution 1:500. The quantitative periodic distance analyses of the images were done using TTorg plugin form ImageJ (Pasqualin et al., 2015).

### Data analysis

Results were expressed as median and interquartile range. Significant differences were determined by ANOVA with Bonferonni *post-hoc* pairwise multiple comparisons vs. the control. Data were box-plotted, where the median is represented by a line and the extremes of the box by 25 and 75% quartiles. The whiskers represent the 95% confidence interval. The images acquired by immunofluorescence were analyzed by ImageJ. All statistical analyses were performed using GraphPad Prism version 5.00 for Windows, GraphPad Software, San Diego California USA, www.graphpad.com.

## Results

### NO negatively modulates force production in skeletal muscle of zebrafish larvae

**Figure 1:**
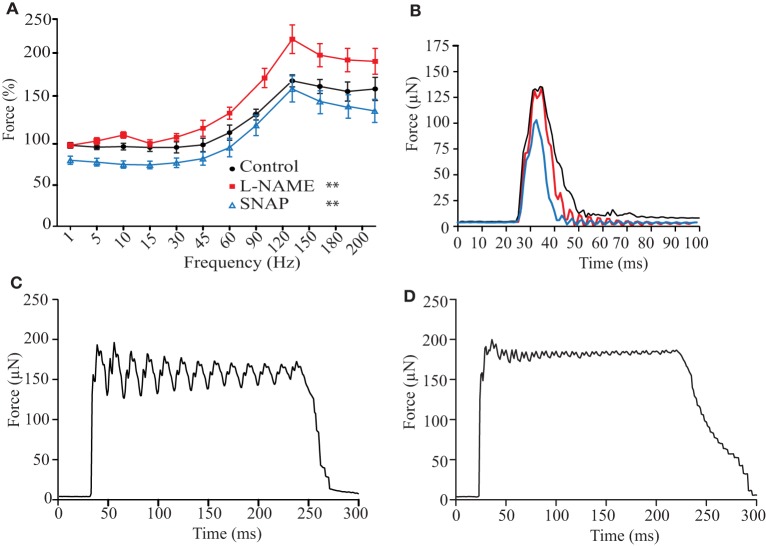
NO negatively modulates the force-frequency relationship in 5–7 dpf zebrafish. The zebrafish was mounted in a force transducer and electrical field stimulation using a pair of platinum electrodes was applied at 200-ms stimulation trains at 2-ms pulse duration and supramaximal voltage, in 2-min intervals. **(A)** Statistical analysis of force-frequency relationship under control (black), 100 μM SNAP (blue), and 5 mM L-NAME (red). Force was normalized to single twitches performed previous to force-frequency measurements in each experiment and the change of force is plotted against stimulated frequency. Representative traces of 1 Hz **(B)**, 120 Hz **(C)**, and 200 Hz **(D)** of electrical stimulation. Values are means ± SD, *n* = 9 animals per group. In comparison to control, the two-way ANOVA analysis showed a significant interaction between frequency and the treatment for L-NAME and SNAP; ^**^p < 0.01.

In order to understand the role of NO on the basic response of muscle contraction of zebrafish, the force-frequency relationship was obtained from electrically stimulated decapitated fish using a voltage-time protocol (18 V, 2 ms during 200 ms every 2 min) in control solution only or with addition of an unspecific NOS blocker (L-NAME) or the NO donor (SNAP) compared to control solution (Figure 1A). Since the standard procedure of normalization using the cross-sectional area was not possible, single twitch force response under control solution and previous the experimental protocols described above was recorded. This value was used to normalized the force-frequency relationship under the effect of SNAP or L-NAME. In general, force production did neither increase steeply with the increase of stimulation frequency, nor was a stable plateau reached at high frequencies of stimulations as observed in mammals. In control solution, normalized force production was around 67% at maximum simulation frequency (Figure 1A). Single twitch stimulation showed a typical response (Figure 1B). Interestingly, we did not observe a fused tetanus at 120 Hz as reported in mammals at these high frequencies of stimulation (Figure 1C), but only at 200 Hz (Figure 1D). The two-way ANOVA analysis showed a significant interaction between frequency and the treatment with L-NAME and SNAP (*F* = 2.22 DF22; 165; *p* < 0.0024). Normalized force production under the effect of L-NAME was not different from control at low and medium stimulation frequency (from 1 to 60 Hz). However, at high frequency of stimulation (90–200 Hz), the force was higher (Figure 1A). In presence of SNAP, the normalized force was lower compared to control from 1 to 60 Hz of stimulation and no difference was noted above 90 Hz. A similar effect was observed with a different NO donor, 1 μM DEA- NONOate, at the concentration that releases similar amounts of NO as SNAP (data not shown).

Further analyses of the biophysical parameters during single twitches (1 Hz) were done summarized in Table 1. Although, several parameters suggested a tendency of change, such as Time to Peak and Peak Area, only Peak and Duration_50_ parameters were significantly reduced with SNAP. These results suggest that SNAP's mechanism of action to reduce force production is reducing the initial contraction; on the other hand, the effect of L-NAME was reducing the biophysical parameters related to uptake (Uptake Time, Slope Fall). These results suggest that NO modulates the force production based on initial contractions and the lack of NO alters relaxation.

**Table 1:** Summary of force biophysical parameters obtained from single twitch electrically stimulated skeletal muscle of zebrafish larvae.

**Biophysical parameter**	**Control**	**SNAP (100 μM)**	**L-NAME (5 mM)**
Duration (ms)	58.3; 29.0–116.8	87.3; 46.0–152.2	30.5; 23.7–70.2
Peak (μN)	129.7; 100.2–167.1	99.7; 76.7–112.5^*^	118.9; 112.0–175.5
Time to peak (ms)	9.50; 6.18–9.80	6.50; 6.10–9.10	8.50; 6.10–9.10
Peak area (μN^*^s)	2.02; 1.23–3.10	1.34; 0.96–2.01	1.40; 2.76–1.32
Duration_50_ (ms)	13.1; 12.0–13.5	10.0; 8.5–12.7^*^	11.9; 11.6–12.6
Tau (ms)	6.76; 5.50–7.98	4.70; 3.78–6.68	5.46; 4.45–7.32
Slope of contraction (μN/s)	14,975; 13,213–19,383	14,100; 10,900–16,200	16,130; 14,770–18,980
Relaxation time (ms)	50.4; 23.6–107.3	78.4; 39.8–144.0	22.0; 15.0–60.7
Slope of relaxation (μN/s)	−6,269; −7,619 to −4,382	−3,910; −8,144 to −2,300	−11,440; −15,770 to −4,550

Values expressed as median; 25–75%.

* *p < 0.05 vs. control*.

### nNOS is expressed in similar periodic distance of DHPR and SERCA1

**Figure 2:**
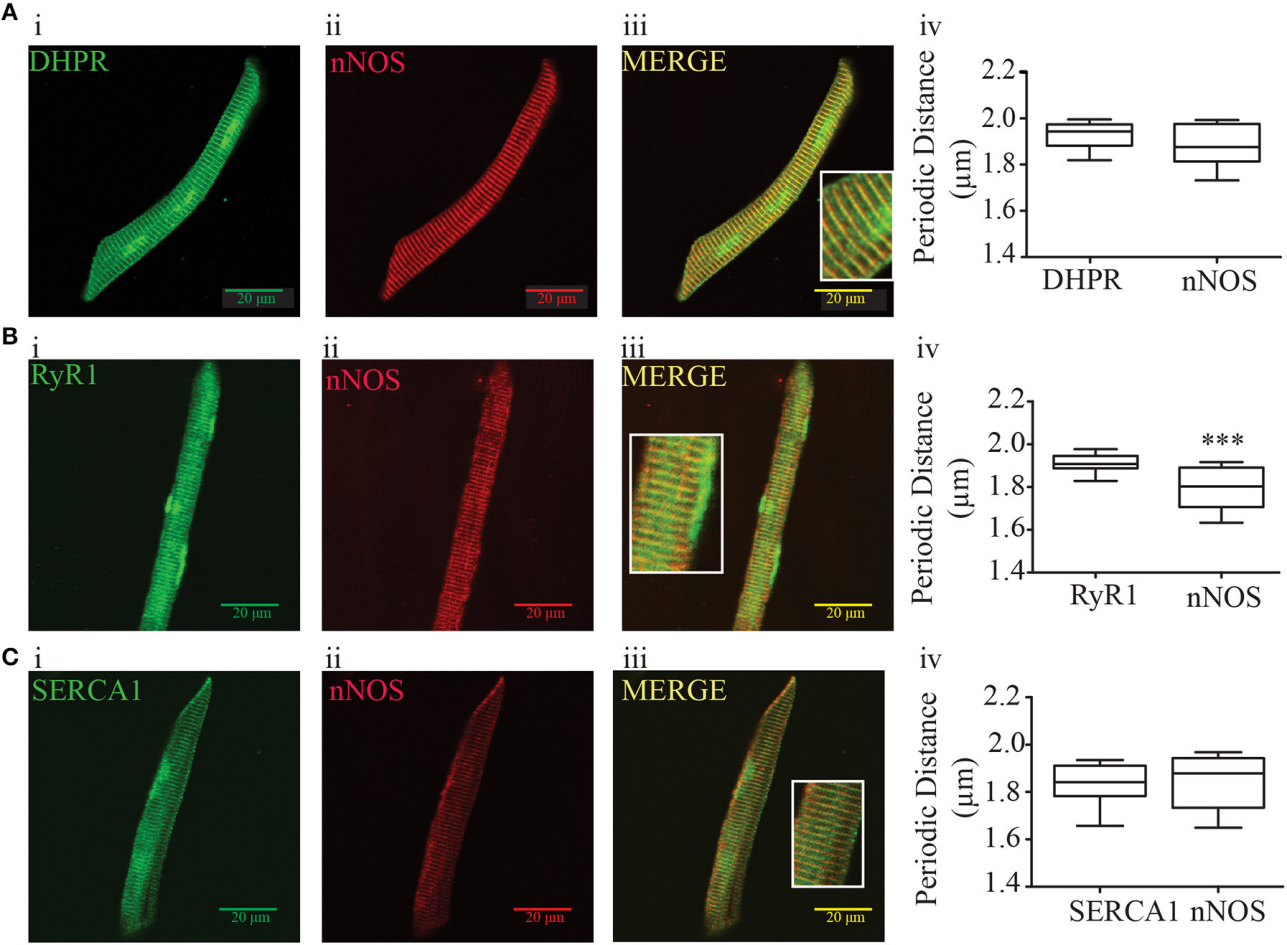
Immunofluorescence and periodic distance analysis of DHPR, RyR, SERCA, and nNOS from zebrafish isolated myocytes. **(A)** Immunofluorescence for DHPR **(i)**, nNOS **(ii)**, and merge **(iii)**, and periodic distance analysis **(iv)** from 26 cells. **(B)** Immunofluorescence for RyR **(i)**, nNOS **(ii)**, and merge **(iii)**, and periodic distance analysis **(iv)** from 18 cells. **(C)** Immunofluorescence for DHPR **(i)**, nNOS **(ii)**, and merge **(iii)**, and periodic distance analysis **(iv)** from 16 cells. The insert at the merged picture shows a magnification of the respective picture. Values are expressed as median; 25–75%. ^***^p < 0.001 vs. RyR immunofluorescence.

Based on the modulatory effect observed with the force-frequency data described above, we mathematically evaluated the spatial expression of nNOS in zebrafish myocyte and correlated it with the expression of DHPR, RyR1, and SERCA1 using TTorg plugin of ImageJ (Figures 2A–C). Confocal analysis of double-staining images of DHPR, RyR1, or SERCA1 with nNOS allowed us to measure the periodic distance of the peak amplitude in the Fourier spectrum of the image taken at the subsarcolemmal region. These analyses support the image data where DHPR and nNOS presented similar periodic distances in the cell (Figure 2Aiv). However, the periodic distance was significantly different comparing RyR1 and nNOS immuno-localization (Figure 2Biv). Finally, there was no difference in the spatial distribution of SERCA1 and nNOS in zebrafish myocyte (Figure 2Civ). These results suggested that nNOS has similar spatial periodicity with DHPR, which is similar to the T-tubule structure and SERCA1 but not the RyR1.

### NO modulates Ca^2+^ transients via the sGC-cGMP pathway

**Figure 3:**
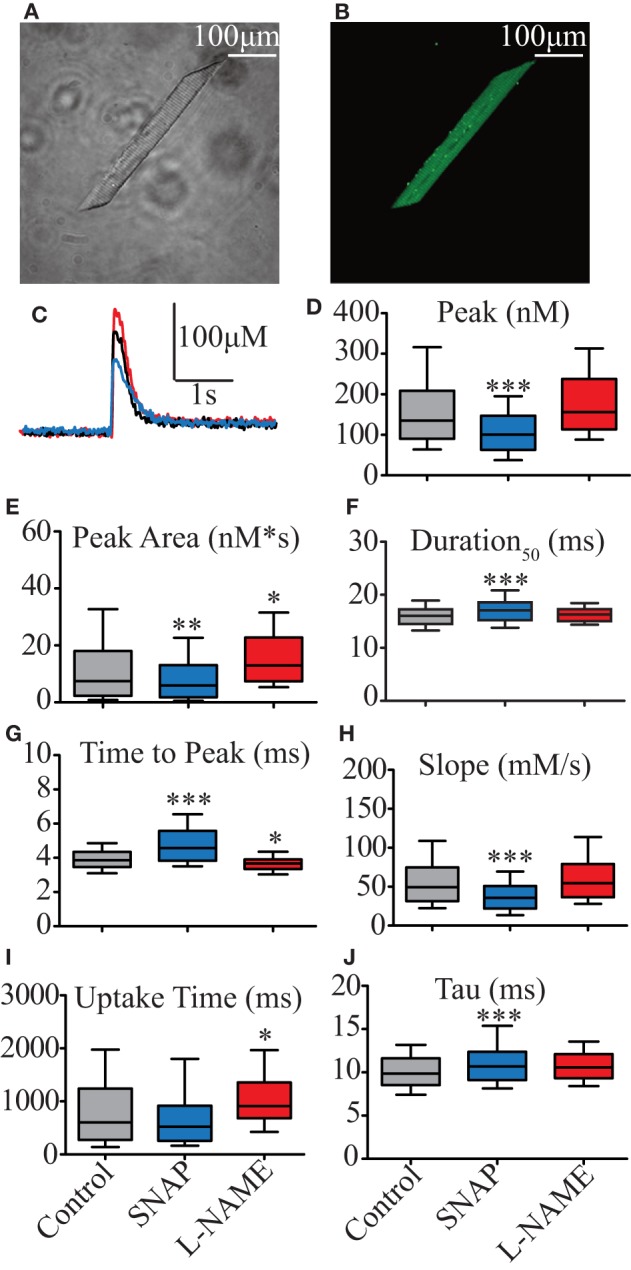
Effect of NO on biophysical parameters of isolated zebrafish myocyte Ca^2+^ transients. **(A)** Photomicrography of isolated zebrafish myocytes under light microscope. **(B)** Photomicrography of the same myocyte shown in **(A)** loaded with 10 μM Fluo4-AM and seen under fluorescence microscope. **(C)** Representative Ca^2+^ transient's traces obtained from electrical field stimulation. Control represented in black, 00 μM SNAP in blue, and 5 mM L-NAME in red. Statistical analyses of Ca^2+^ transients' biophysical parameters: **(D)** Peak (nM), **(E)** Duration_50_ (ms), **(F)** Peak Area (nM^*^s), **(G)** Time to Peak (ms), **(H)** Slope (μM/s), **(I)** Uptake Time (ms), and **(J)** Tau (s). Values are expressed as median; 25–75%. ^*^p < 0.05, ^**^p < 0.01, ^***^p < 0.001 vs. control; *n* = 278 cells for control, 153 cells for SNAP and 106 cells for L-NAME.

Force production is directly dependent on Ca^2+^ homeostasis (Ca^2+^ release and uptake) during each contraction. Therefore, the analyses of Ca^2+^ transients from isolated skeletal muscles provide evidence of the modulatory effect of NO. The size of a typical isolated skeletal myocyte from zebrafish larvae was ~15 × 100 μm, similar to a rectangular shape (Figure 3A). The striation characteristics of the sarcomere formation were clearly visible in transmission light, similar as described in adult human muscles (Figure 3A). The diffusion of Fluo-4 was even in the entire myocyte and the background intensity was constant during the recording of Ca^2+^ transients (Figure 3B). The isolated myocytes maintained these characteristics of size and shape over the course of the experiments, while movement artifacts were avoided using low concentrations of BDM in the plating medium (10 mM), but it was absent in working solutions. Table 2 summarize the values of 7 parameters established for Ca^2+^ transients' analyses, where three describe the general aspects of Ca^2+^ transients (Peak, Duration_50_, and Peak Area), two Ca^2+^ release (Time to Peak and Slope) and two Ca^2+^ uptake (Uptake Time and Tau). As shown in Figure 3C, the NO donor SNAP negatively modulates the Ca^2+^ transient, whereas the presence of L-NAME induces a slight enhancement. Compared to control, 100 μM SNAP significantly reduced Peak (Figure 3D) and consequently a significant reduction in the Peak Area (Figure 3E). The reduced NO-mediated Ca^2+^ transient increased Duration_50_ (Figure 3F) and Time to Peak (Figure 3G), with a reduction in Slope (Figure 3H). Ca^2+^ uptake was not significantly reduced (Figure 3I), but Tau was significantly increased (Figure 3J). Conversely, 5 mM L-NAME did not significantly increase either Peak or Duration_50_s, but it increased Peak Area (Figure 3F). Even with a significant reduction of Time to Peak (Figure 3G), the increase of Peak Area was mainly due to a significant increase of Uptake Time (Figure 3I). These results suggested that NO is able to modulate the Ca^2+^ homeostasis of isolated zebrafish myocytes during single twitch stimulation. These results agree with the single twitch force analysis described above where SNAP modulated the force production via Ca^2+^ release and L-NAME modified Ca^2+^ uptake.

**Table 2:** Summary of NO effect on Ca^2+^-transient biophysical parameters obtained from electrically stimulated isolated myocytes of zebrafish larvae.

**Biophysical parameter**	**Control**	**SNAP (100 μM)**	**L-NAME (5 mM)**
Duration (ms)	608.4; 277.4–1,245	528.7; 261.4–922.4	900.8; 590.3–1,333
Peak (nM)	135.1; 90.1–208.7	100.6; 62.9–147.2^**^	156.2; 113.4–237.8^*^
Time to peak (ms)	3.86; 3.46–4.35	4.57; 3.83–5.57^***^	3.66; 3.35–3.90^*^
Peak area (nM^*^s)	7.47; 2.30–18.0	5.95; 1.78–13.0	12.9; 7.39–22.75
Duration_50_ (ms)	16.1; 14.5–17.3	17.1; 15.2–18.6^***^	16.3; 15.0–17.3
Tau (ms)	9.86; 8.53–11.6	10.7; 9.11–12.4^***^	10.6; 9.32–12.1
Slope of release (μM/s)	49.3; 31.7–74.7	35.6; 21.9–50.9	54.6; 36.43–79.0
Uptake time (ms)	604.4; 272.6–1,241	521; 255.9–918.2	897.1; 681.0–1356^*^

Values expressed as Median; 25–75%.

* p < 0.05;

** p < 0.01,

*** *p < 0.001 vs. control*.

Interestingly, we observed that during electrical stimulation the fluorescence produced by Fluo4 during the Ca^2+^ transient was not uniform in the entire isolated myocyte, independent of the concentration of Fluo4, time of incubation, temperature, or the presence of NO donor or of NOS blocker. In order to study whether the changes of intensity of fluorescence related to the Ca^2+^ transient might occur in the entire myocyte or in defined areas, we electrically stimulated isolated myocytes and measure Ca^2+^ transients in laser line scanning mode on a confocal microscope. The changes of Ca^2+^-mediated fluorescence were uniform across the myocyte at the subsarcolemmal region, arbitrarily set as 0 μm of the myocyte (Figure S1A). However, when the laser scanning was set into the myocyte in 1 μm steps, the changes of Ca^2+^-mediated fluorescence was only detected from the edge until the center of the cell (Figures S1B–F). This result showed that the Ca^2+^ transients in large parts occur underneath the surface of the sarcolemma, suggesting that at this age the isolated myocyte of the zebrafish is still immature and cytosolic components are probably still developing, which would explain the limited force production described above.

**Figure 4:**
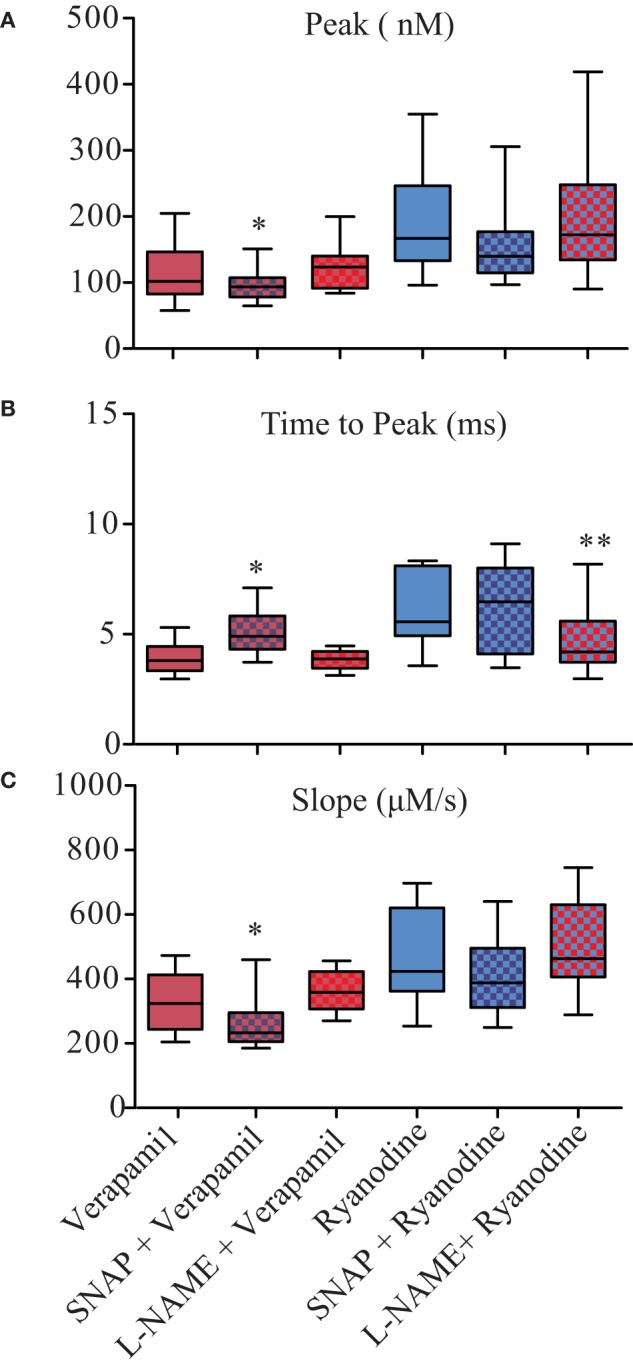
NO negatively modulates Ca^2+^ release biophysical parameters of isolated zebrafish myocytes. Statistical analyses of Ca^2+^ release parameters from electrically field stimulated isolated myocytes under SNAP (100 μM) or L-NAME (5 mM) in combination with verapamil (20 μM) or ryanodine (100 nM). **(A)** Peak (nM), **(B)** Time to Peak (ms), and **(C)** Slope (μM/s). Values are expressed as median; 25–75%. ^*^p < 0.05, ^**^p < 0.01, vs. verapamil or ryanodine; *n* = 38 cells for verapamil, 17 cells for Verapamil + SNAP, 18 cells for Verapamil + L-NAME, 34 cells for Ryanodine, 17 cells for Ryanodine + SNAP, and 15 cells for Ryanodine + L-NAME.

The analyses of the biophysical parameters of single twitches and Ca^2+^ transients suggested that SNAP would modulate the Ca^2+^ release and L-NAME the Ca^2+^ uptake. Therefore, we evaluated the effect of NO on Ca^2+^ release in isolated myocytes using classical pharmacological tools of excitation-contraction coupling (EC-coupling). First, we blocked the DHPR with verapamil (20 μM) in presence of SNAP or L-NAME. Compared to control conditions, verapamil radically suppressed the Peak of Ca^2+^ transients (Figure 4A). This effect was slightly enhanced in presence of exogenous NO generated by SNAP, where Peak (Figure 4A) and Slope (Figure 4C) were reduced and Time to Peak (Figure 4B) increased. Compared to control, these parameters were unaltered in presence of L-NAME (Table S1).

Since high concentrations of ryanodine (2 μM) completely blocked Ca^2+^ transients (data not shown), we locked RyR1 at a half open stage with lower ryanodine concentration (100 nM) in presence of SNAP or L-NAME (Figure 4). We observed that the effect of lower concentrations of ryanodine on Ca^2+^ release parameters were higher compared to verapamil, but still reduced compared to control solution (Table S1). Both SNAP and L-NAME did not produce a significant change in the Peak of Ca^2+^ transients in combination with ryanodine (Figure 4A), except the combination of L-NAME with ryanodine did reduce the Time to Peak (Figure 4B). To further evaluate the role of NO on Ca^2+^ release, we electrically stimulated the myocytes under the effect of caffeine (250 μM) as an activator of RyR1. At this concentration, the Peaks were significantly higher compared to control solution (Table S1). This enhanced response of the Ca^2+^ release peak was also modified by SNAP; SNAP significantly reduced Peak and Slope, while the presence of L-NAME did not alter any of the analyzed parameters. Altogether, these results suggested that exogenous NO negatively modulates the DHPR, which consequently would reduce the Ca^2+^ release via RyR from the SR in zebrafish larvae skeletal muscle.

**Figure 5:**
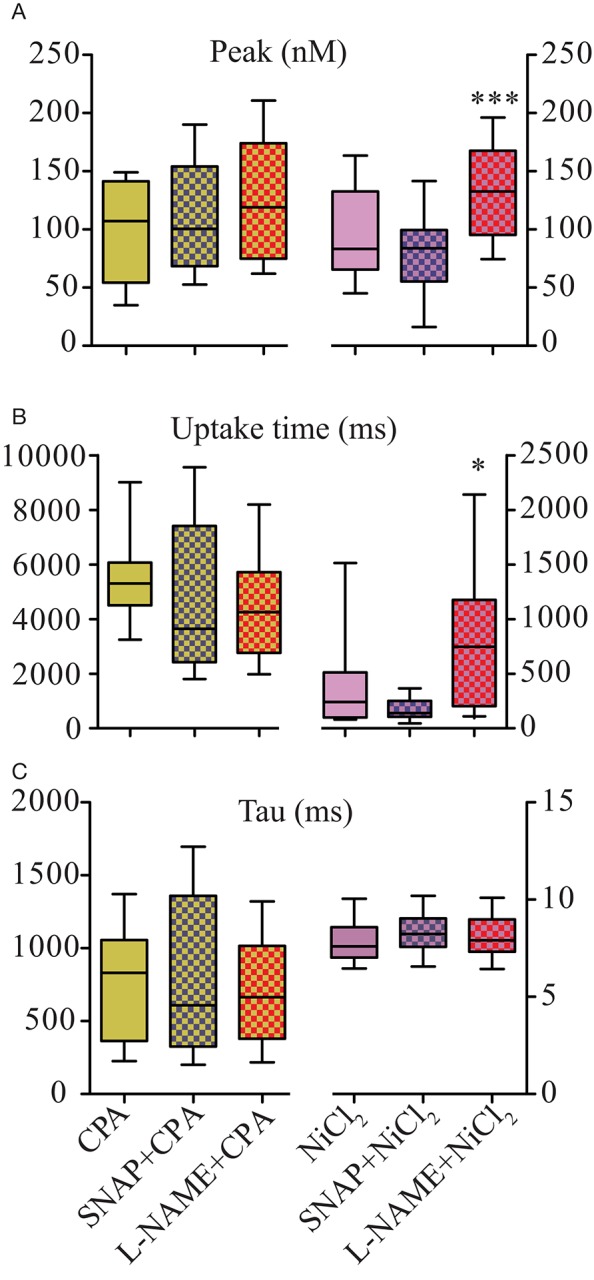
SERCA Ca^2+^ uptake biophysical parameters of isolated zebrafish myocytes were negatively modulated by NO. Statistical analyses of Ca^2+^ release parameters from electrically field stimulated isolated myocytes under SNAP (100 μM) or L-NAME (5 mM) in combination with CPA (2.5 μM) on the left or NiCl_2_ (2 mM) on the right. **(A)** Peak (nM), **(B)** Uptake Time (ms), and **(C)** Tau (s). Values are expressed as median; 25–75%. ^*^p < 0.05; ^***^p < 0.001, vs. NiCl_2_; *n* = 19 cells for CPA, 14 cells for CPA + SNAP, 16 cells for CPA + L-NAME, 54 cells for NiCl_2_, 22 cells for NiCl_2_ + SNAP and 19 cells for NiCl_2_ + L-NAME.

The results shown in Figure 3 suggest that NO also modulates Ca^2+^ uptake in Ca^2+^ transients. In order to address this question, we evaluated two major Ca^2+^ uptake mechanisms: SERCA and NCX. We evaluated the role of NO on NCX function blocking SERCA with CPA (2.5 μM). The analysis of the Peak parameter did not show any substantial change attributable to NCX (Figure 5A); however, the analysis on Uptake Time and Tau showed an enormous difference compared to control solution (Table 2 vs. Table S2), suggesting a remarkable importance of SERCA in the Ca^2+^ removal in skeletal muscle of zebrafish larvae (Figure 5B). In presence of CPA, the Uptake Time was 6-fold higher and Tau was ca. 10-fold higher compared to control solution. The presence of SNAP or L-NAME did not significantly change either Uptake Time or Tau (Figures 5B,C). These results support previous evidences that SERCA is the main mechanism of cytosolic Ca^2+^ removal and NO did not significantly modify NCX activity.

The role of SERCA on Ca^2+^ removal was studied blocking NCX with NiCl_2_ (2 mM). As expected, release parameters of myocytes treated with NiCl_2_ were similar to control solution, showing that at this concentration NiCl_2_ did not interfere with DHPR or RyR activities. SNAP in combination with NiCl_2_ did not modify the Peak parameters (Table S2); however, L-NAME in combination with NiCl_2_ significantly increased the Peak parameters (Figure 5A). Compared to NiCl_2_ solution alone SERCA-dependent Ca^2+^ removal time was significantly reduced in solutions containing NiCl_2_ and SNAP, probably due to a reduction of the Ca^2+^ transient duration. Reversely, SERCA-dependent Ca^2+^ was not significantly increased in presence of NiCl_2_ and L-NAME (Figure 5B). However, Tau was similar under all three conditions (Figure 5C), suggesting that NO did not modify cytosolic SERCA-dependent Ca^2+^ removal.

**Figure 6:**
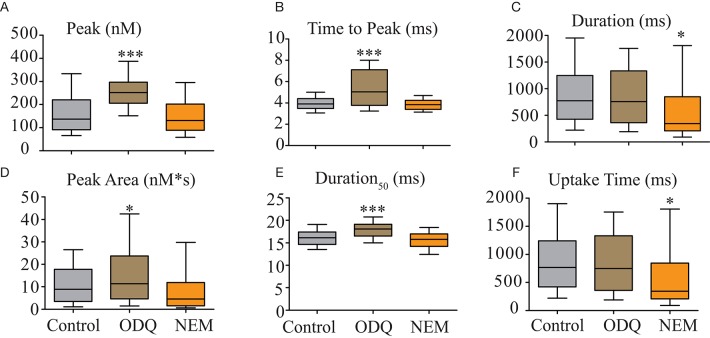
NO modifies Ca^2+^ transients biophysical parameters via sGC pathway. Statistical analyses of Ca^2+^ transients' parameters from electrically field stimulated isolated myocytes under ODQ (10 μM) or NEM (100 μM). **(A)** Peak (nM), **(B)** Time to Peak (ms), **(C)** Duration (ms), **(D)** Peak Area (nM^*^s), **(E)** Duration_50_, and **(F)** Uptake Time (ms). Values are expressed as median; 25–75%. ^*^p < 0.05, and ^***^p < 0.001 vs. control; *n* = 178 cells for control, 100 cells for ODQ and 100 cells for NEM.

To evaluate the mechanism by which endogenous NO modulates Ca^2+^ transients in zebrafish myocytes, we used two pharmacological tools: a sGC inhibitor, ODQ (10 μM) in order to evaluate the effect mediated by sGC and the S-nitrosylation blocker NEM (100 μM) to block the effect mediated by S-nitrosylation. Results are shown in Figure 6 and summarized in Table 3. In general, the use of ODQ enhanced the effect of L-NAME, while NEM presented little or no effect on most of the parameters. ODQ significantly increased Ca^2+^ transient peak (Figure 6A), time to peak (Figure 6B), peak area (Figure 6D) and duration_50_ (Figure 6E). NEM reduced duration (Figure 6C) and uptake time (Figure 6F). The effect of ODQ is similar to what was described above for L-NAME, suggesting that the major effect of endogenous NO on biophysical parameters of Ca^2+^ uptake during Ca^2+^ transients is via sGC in 5–7 days old zebrafish skeletal muscle.

**Table 3:** Summary of NO-sGC and S-nitrosylation effects on Ca^2+^-transient biophysical parameters obtained from electrically stimulated isolated myocytes of zebrafish larvae.

**Biophysical parameter**	**Control**	**ODQ (10 μM)**	**NEM (100 μM)**
Duration (ms)	772.3; 428.3–1246	755.9; 362.6–1335^*^	346.1; 209.3–847.5
Peak (nM)	136.8; 90.5–220.4	252.1; 206.1–297.4^***^	130.7; 88.8–201.8
Time to peak (ms)	3.90; 3.47–4.40	5.03; 3.76–7.11^***^	3.83; 3.40–4.23
Peak area (nM^*^s)	8.90; 3.46–17.8	11.4; 4.65–23.8^*^	4.57; 1.52–11.9
Duration_50_ (ms)	16.1; 14.7–17.4	18.07; 16.5–19.1^***^	15.8; 14.2–17.0
Tau (ms)	9.7; 8.5–11.5	11.93; 10.5–14.2^***^	10.5; 9.10–11.8
Slope of release (μM/s)	49.7; 31.2–77.9	55.5; 36.7–84.3	42.4; 26.1–69.8
Uptake time (ms)	766.8; 421.4–1241	749.4; 358.4–1332	342.4; 205.2–843.5^*^

ODQ (10 μM) is sGC blocker and NEM (100 μM) is a blocker of S-nitrosylations. Values expressed as Median; 25–75%.

* p < 0.05;

*** *p < 0.001 vs. control*.

## Discussion

Here we present evidence that in zebrafish larvae endogenous NO reduces force production by reducing Ca^2+^ release in a sGC-dependent pathway. Force production was negatively modulated by exogenous NO and enhanced in absence of NO produced by endogenous NOS. Although it is known the NO decrease myofibrillar Ca^2+^ sensitivity (Andrade et al., 1998), here we focused to further elucidate the inhibitory effect of NO on Ca^2+^-dependent force production. Different to Andrade et al. (1998), we (1) used a detailed method to describe different aspects of force and Ca^2+^ transients we collectively termed biophysical parameters, (2) we analyzed our data based on single twitch stimulation and not from submaximal tetanic stimulation, (3) we used L-NAME to evaluate the endogenous effect of NO on muscle contraction and on Ca^2+^ transients, and (4) we used zebrafish larvae as an animal model. These differences allowed us to quantify in detail each aspect of NO-dependent force and Ca^2+^ transient changes, measuring the effect of endogenous NO on force and Ca^2+^ transients in a well-established animal model for patho-physiological muscular approaches that have been extensively used due to easy and fast production of mutants, helpful model to test new therapeutic strategies and low-cost maintenance (Dou et al., 2008; Telfer et al., 2012; Asaduzzaman et al., 2013; McGown et al., 2013; Li et al., 2014; Cho et al., 2015; Li and Arner, 2015).

Since it was not possible to calculate the cross sectional area for normalization of force production as performed in other muscle models, we found that in our model single twitch force at 1 Hz prior to the force-frequency protocol was suitable for normalization to standardize the results obtained from different sizes of zebrafish larvae. The increase of force production across the entire force-frequency protocol revealed that larvae did not produce a sigmoidal force increase in zebrafish (Martin et al., 2015) as observed in mammalian skeletal muscle (Kobzik et al., 1994; Reid, 1998; Reid et al., 1998). Although we followed the force production protocol published by Dou et al. (2008), our force was substantially smaller than expected. The procedure to hold the zebrafish larvae in the aluminum foil was extremely difficult and our rate of success was very low. Therefore, we adapted the method using cyanoacrylate to better fix the larvae in the aluminum foil. Although, being very cautious we were not able to record from the entire fish as shown in Figure 1 by Dou et al. Having less muscle tissue, we expected to measure smaller forces in comparison to Dou et al. In fact, our data showed small variation and validated that our modified procedure described above did not cause major differences among the samples from the same group. Nevertheless, the maximal force production was ca. 67% higher compared to single twitch stimulation, which is in line with previous reports for zebrafish force-frequency (Dou et al., 2008). Additionally, the zebrafish larvae adapted to our method was also able to respond to extreme high frequencies of stimulation (120 Hz) without showing the clear characteristics of a fused tetanus, only at 200 Hz. This result is in line with the previous report by others (Dou et al., 2008; Martin et al., 2015), whose protocols reported that complete fused tetanus was obtained at high frequency stimulation of ~180 Hz. Attili and Hughes (2014) also field-stimulated zebrafish larvae at 200 Hz in order to evoke muscle contractions to evaluate the effect of tricaine measuring rapid escape movements. As described by Attili and Hughes, zebrafish larvae would not be able to move upon stimulation at 200 Hz (Attili and Hughes, 2014), but did upon single twitch stimulation (Attili and Hughes, 2014; Tabor et al., 2014). The physiological relevance of the force-frequency data relies on the frequency of the tail used by the zebrafish for swimming. Zebrafish larvae swim in discrete bouts with multiple oscillations of the tail, inter-spaced by quiescent periods (Budick and O'Malley, 2000) and the swimming movement of the tail is a synchronized process where motor neurons activated the correct amount of muscles fibers to produce the necessary force for a precise response, i.e., swimming for escaping, mating or preying. The measured frequency of tail's muscles during spontaneous or induced activity ranged between 15 and 100 Hz, depending on age, experimental set-up and genotype (Budick and O'Malley, 2000; Buss and Drapeau, 2001; Muller and van Leeuwen, 2004; McDearmid and Drapeau, 2006; Portugues and Engert, 2011). From this range, we observed that 60 and 90 Hz are the frequencies of stimulations able to produce the maximal force that the zebrafish larvae could generate for swimming. However, we cannot extrapolate the single twitch data analyses (that it is discussed below) to higher frequency of stimulation due to: (1) tetanic stimulation requires higher frequency and longer time compared to single twitch that modifies the EC-coupling, such as a presence of slow calcium entry during tetani stimulation that is refractory to ryanodine, but it is IP3-dependent (Eltit et al., 2004). Moreover, it is difficult to predict the role of NO under sub tetanic or tetanic conditions; and (2) we observed from the force-frequency data that the force produced at any sub-tetanic or tetanic frequency of stimulation is not a factor of the 1 Hz data (it is not lineal). Despite these limitations, we considered that our aim in this study was achieved using classical single twitch EC-coupling methods to reveal in detail the effect of NO on zebrafish larvae.

The requirement to produce force before fusion into a tetanus begins can be explained with an extreme fast action potential and fast Ca^2+^ homeostasis (Buckingham and Ali, 2004; Coutts et al., 2006). This interesting characteristic was previously reported, where the half-duration of recorded action potentials was as fast as <0.6 ms (Coutts et al., 2006). Moreover, the kinetics and the steady-state characteristics of the K^+^ and Na^+^ currents of inner skeletal muscles allow high frequency firing expected during fast swimming (Buckingham and Ali, 2004; Coutts et al., 2006). Although, both zebrafish's force production and Ca^2+^ homeostasis have been characterized, there is no information regarding the effect of NO on force and Ca^2+^ transients. We observed that exogenous NO produced by 100 μM SNAP was enough to reduce the force production at low frequencies of stimulation. We used SNAP at the concentration of 100 μM, which according to the manufacturer and our previous experience would produce NO at ca. 1.4 μM (Iturriaga et al., 2000a,b). Furthermore, the reduction of force production at low frequencies was confirmed using DEA- NONOate, a different NO donor. Conversely, blocking NOS activity with L-NAME induced an increase of force production only at very high stimulation frequency. A similar effect was extensively reported in various mammalian skeletal muscles (Kobzik et al., 1994; Andrade et al., 1998; Reid, 1998).

Based on results described above, which confirm those previously obtained in mammalian models that NO negatively modulates force production, we evaluated whether the effect of NO on force production could be due to modification of Ca^2+^ transients. To test this hypothesis, we isolated the skeletal muscle from zebrafish larvae and loaded them with Fluo4 Ca^2+^ indicator. Although, we used a different method, we obtained similar size and shape of isolated skeletal muscles as described by Robin and Allard (2015). However, we observed a difference in sarcomere length compared to reported by Dou et al. where they measured the change of sarcomere length during electrical stimulation using a laser beam method in alive zebrafish tissue while is still fixed in the force transducer. In our case, the sarcomere length was measured in single cells after the immunostaining protocol using the fluorescence signal from three different proteins (DHPR, RyR, and SERCA). If it assumed that the single cell is relaxed (1.9 μm) as opposed to the electrically stimulated mounted muscle in the force transducer, the sarcomere lengths seem comparable. Next, we evaluated whether nNOS would have a similar periodic distance as DHPR, RyR, and SERCA. The double immuno-fluorescence images suggested that the nNOS signal would be distributed similarly to DHPR, RyR1, and SERCA. However, our confocal microscope allowed us to have a maximal resolution of 440 nm at lateral direction (xy dimension) and 1.62 μm at axial direction (z dimension) for both 488 and 650 nm wavelengths necessary to excite the second fluorescence antibodies (Georgiev et al., 2015). Therefore, interpretation of co-localization of nNOs with the other tested proteins has to be done with care. In order to avoid misinterpretation of our immune-fluorescence data, we used a mathematical approach using TTorg plugin for ImageJ, which measures the periodic distance of the peak amplitude in the Fourier spectrum of the image, determining whether nNOS would have the same pattern of distribution (periodic distance) of DHPR, RyR, and SERCA. The analysis from the quantification obtained by the TTorg plugin showed only a significant difference in the periodic distance of nNOS respect to RyR. This result suggested that nNOS at 5–7 dpf larvae zebrafish is not distributed randomly in the sarcolemma and presents a similar periodic distance measured for DHPR and SERCA. It is interesting to note that we observed the Ca^2+^ transients were not uniformly distributed across the entire myocyte. They were rather more intense at the subsarcolemmal region (Figure S1). Therefore, we further investigated measuring the Ca^2+^ transient using line-scan recordings on a confocal microscope and to clarify whether the Ca^2+^ transients were limited to the salcolemmal or it also happened into the myocyte. We observed that in 5–7 dpf, the Ca^2+^ transients occurred starting in the subsarcolemmal region until approximately the center of the myocyte. Previous electron microscopy data showed that t-tubules seem to be well formed in 7 dpf (Zhang et al., 2009; Gupta et al., 2011; Housley et al., 2016). However, Gupta et al. (2011), showed that both RyR and DHPR are present at the outermost edge of the cell (Figure 7K, p. 1,720) and only the RyR is present at the sarcolemma, supporting our observations regarding Ca^2+^ transients described above.

The kinetics of Fluo4 Ca^2+^ transients were slower compared to the previously described Ca^2+^ transients obtained using whole cell patch clamp technique. However, this trait, of Fluo4 Ca^2+^ transients was useful for evaluation of the individual biophysical parameters of the modulatory effect of NO in zebrafish larvae from a selected region of the isolated myocyte. In addition, we successfully used the same method described here were in isolated zebrafish cardiomyocytes (Scheid et al., 2016). Moreover, the possibility to record several cells per experiment allowed us to statistically analyze a large number of cells compared to the patch clamp technique obtaining data. Our analyses of the biophysical parameters of single twitch-evoked Ca^2+^ transients showed that exogenous NO significantly reduced the Peak and Peak Area relative to control solution, which supports the finding that NO significantly reduced force production. In detail, the analyses of other parameters such as Time to Peak and Slope suggested that exogenous NO significantly prolonged the time of Ca^2+^ release with a decreasing rate of release, which results in a smaller Ca^2+^ transients, which in turn is translated to reduced force production. Conversely, blocking NOS activity enhanced the Peak of Ca^2+^ transients and significantly increased Peak Area compared to control. Moreover, Time to Peak was significantly reduced and the Slope was increased, but this was not statistically significant. These results suggest that NO modulates crucial events of Ca^2+^ transients such as Ca^2+^ release and Ca^2+^ uptake. To further answer this question, we tested the effect of NO on Ca^2+^ release and uptake in isolated myocytes using traditional pharmacological tools. The analyses from blocking DHPR with 20 μM verapamil showed that in combination with either exogenous NO or blocking NOS the Ca^2+^ transients displayed similar behavior regarding Peak, Time to Peak and Slope as observed from addition of SNAP or L-NAME without verapamil (Figure 4). These results support previous reports showing that the DHPR is not responsible for Ca^2+^ conductance but rather for the activation of the RyR1 in order to activate the EC-coupling via voltage-dependent conformational change of the β_1a_ subunit (Schredelseker et al., 2009, 2010).

We next evaluated the role of RyRs on Ca^2+^ transients under the effect of NO using 100 nM of ryanodine or 250 μM caffeine. The observed effect of exogenous NO (SNAP) on the biophysical parameters of Ca^2+^ transients were absent once SNAP was simultaneously applied with 100 nM ryanodine. At high ryanodine concentrations, the RyRs were completely blocked and it was no possible to elicit Ca^2+^ transients even at higher or longer electrical stimulation. However, at 100 nM ryanodine, the RyRs are locked at a subconductance state and do not open to a full conductance state (Koulen et al., 2001). Taken together, this suggests that exogenous NO released by SNAP would not modulate RyR activity. Conversely, the results of L-NAME (blockage of endogenous NO production) in combination with ryanodine was similar to those obtained without ryanodine, suggesting that endogenous NO would modulate the activity of RyRs. Nevertheless, these results should be treated with caution due to the high variability caused by the constant open subconductance of the RyRs (Koulen et al., 2001). Similar experiments were carried out using 250 μM caffeine as an activator of RyRs obtaining Ca^2+^ transient results comparable to the experiments without caffeine (Table S1). This suggested that under the effect of caffeine, only exogenous NO negatively modulates RyR activity. Zebrafish skeletal muscles express almost equal amounts of both RyR-α and RyR-β types (Ottini et al., 1996; Koulen et al., 2001), which are homologs to the mammalian RyR type 1 and 3, respectively (O'Brien et al., 1995; Ottini et al., 1996; Sonnleitner et al., 1998). Anatomically RyR-α is expressed in the junctional feet where it interacts with the DHPR, forming the triad in skeletal muscle, whereas the RyR-β is expressed in the parajunctional feet, which is located next to the triad facing toward the myofibrils rather than toward the t-tubule (Perni et al., 2015). Both RyR-α and RyR-β show different conductance and Ca^2+^ mediated inhibition: although both RyR-α and RyR-β are activated by physiological low Ca^2+^ concentration, RyR-α has higher conductance and Ca^2+^ inhibition at submillimolar free cytosolic Ca^2+^, whereas RyR-β has lower conductance and is active at millimolar Ca^2+^ concentration (Koulen et al., 2001). Another functional difference between RyR-α and RyR-β is that the first does not respond with Ca^2+^ sparks upon caffeine stimulation, while the second does (Perni et al., 2015). While the role of RyR-α is well-established in zebrafish skeletal muscle, the relevance of RyR-β is still being discussed, where it has been suggested to be part of the calcium-induced-calcium release mechanism and that it is not essential for normal movement of the tail in zebrafish morfants for RyR-β (Perni et al., 2015). In the 1B5 myogenic cell line both RyR1 and RyR3 have the same affinity to ryanodine, but electrical stimulation and 4-chloro-m-cresol are able to induce Ca^2+^ transients in RyR1-expressing cells, while the same protocol does not produce a response in RyR3-expressing cells. Moreover, the application of caffeine induces Ca^2+^ oscillations in RyR1-expressing 1B5 myotubes whereas the same concentration generates a small, localized and sustained Ca^2+^ increase that does not propagate and resolves rapidly in RyR3-expresing cells. Here we were interested in the general response of the RyR to NO and thus, we did not differentiate the contribution of RyR-α and RyR-β on the Ca^2+^ transients, neither whether the effect of NO was equal or not on both RyRs.

Based on the results obtained above (Figure 3), we investigated whether the presence of SNAP or L-NAME would modulate cytosolic Ca^2+^ removal (Figure 5). In mammalian skeletal muscle NO inhibits SERCA activity in a dose-dependent manner in both fast and slow muscles (Kobzik et al., 1994; Ishii et al., 1998) and it has been suggested that NO inhibit SERCA via GMP-PKG pathway (Bredt et al., 1990) or via direct action of NO on SERCA (Ishii et al., 1998); however, the mechanism of action is not entirely clear. Another discussed possibility is that NO inhibits transient receptor potential Ca^2+^ channel isoforms 3 and 6, decreasing Ca^2+^ influx and thus decreasing or depleting Ca^2+^ stores in the SR and consequently inhibit muscle contraction (Ishii et al., 1998; Francis et al., 2010); we did not evaluate this hypothesis due to its complexity and extension of the experiments. Here we pharmacologically evaluated the role of SERCA and NCX, only the most prominent Ca^2+^ extruders of skeletal muscle (Gehlert et al., 2015). We used CPA, a SERCA blocker, in order to evaluate the contribution of NCX to Ca^2+^ uptake. As expected, time durations of Ca^2+^ transients were extremely long under CPA treatment, without a change in the amplitude of the Peak. Under the effect of CPA, the evaluated Ca^2+^ uptake measuring uptake time and tau parameters did not show a significant change with either SNAP or L-NAME. When the myocytes were incubated with the NCX-blocker NiCl_2_ (Gonzalez-Serratos et al., 1996; Deval et al., 2000, 2002a,b; Torok, 2007) the measured SERCA-mediated Ca^2+^ removal showed no significant reduction in uptake time parameter with SNAP (due to a non-significant reduction on the total duration of Ca^2+^ transient). Tau parameter was not different from NiCl_2_ solution with either SNAP or L-NAME. It is interesting to note that higher NiCl_2_ concentrations inhibit calcium transients, possibly because it blocked calcium release. We observed that only at 2 mM there was no such interference of NiCl_2_. These results suggest that SERCA is the main cytosolic Ca^2+^ removal protein in zebrafish myocytes, while NCX showed no measurable effect. Regarding the effect of NO on cytosolic Ca^2+^ removal, there were no significant differences in the tau parameter, suggesting that neither endogenous nor exogenous NO would modulate either NCX- or SERCA-dependent Ca^2+^ uptake.

The NO action on Ca^2+^ transients described above could be mediated by NO-sGC pathway or via S-nitrosylation mechanism. In order to clarify this question, we analyzed the alterations of our proposed biophysical parameters under the effect of the sGC blocker, ODQ (10 μM). Moreover, we analyzed the effect of the S-nitrosylation blocker NEM (100 μM) on the same biophysical parameters. We observed that the sGC blockage with ODQ produced a similar effect as observed with L-NAME, suggesting that the effect of endogenous NO could be via the NO-sGC pathway. We wanted to exclude that this effect could also be related to NO-sGC-independent mechanisms evaluating whether the S-nitrosylation could contribute to the NO-mediated alterations of the biophysical parameters of the calcium transient. We observed that NEM, an S-nitrosylation blocker did not differ from control solution, except for Duration and Uptake time suggesting that S-nitrosylation via endogenous NO was not required for normal Ca^2+^ homeostasis. These two sets of data allowed us to propose that the main effect of NO on Ca^2+^ transients of 5–7 days old zebrafish skeletal muscle is via the NO-sGC pathway.

In summary, our results support previous reports of the NO effect in mammalian skeletal muscle (Kobzik et al., 1994; Andrade et al., 1998; Ishii et al., 1998) and extend them to 5–7 dpf zebrafish skeletal muscle. Our data suggested that NO negatively modulates force production reducing Ca^2+^ release from the SR via NO-sGC pathway, an effect probably mediated by protein kinase G (PKG) since this effect was antagonized by the action of NOS inhibitor or blocker of sGC. The next step would be to evaluate the targets and how PKG activation is able to decrease Ca^2+^ transient and thus force production in zebrafish skeletal muscle. Furthermore, in light of our results it would be of future interest to address the role of NO in other muscle pathways such as PKG activation as seen in skeletal muscle myofilaments (Beckendorf and Linke, 2015) and cardiac muscles (Francis et al., 2010), in the inhibition of the RyR (Aghdasi et al., 1997). The implication of understanding the NO-sGC-PKG pathway would help to design new therapeutic strategies in muscle diseases, such as Duchenne muscular dystrophy, where the correct localization and expression of nNOS is impaired, being replaced by the expression of iNOS hindering correct function of NO (Mosqueira et al., 2013).

## Author contributions

ZX and MM: Contributed with design and performing all experiments, acquisition, analysis, and interpretation of data presented in the manuscript. He also drafted the manuscript and gave his final approval of the version to be published, agreeing with all aspects of the manuscript. RF: Contributed with interpretation of data for the work, drafting the manuscript and final approval of the version to be published and agreed with all aspects of the manuscript.

### Conflict of interest statement

The authors declare that the research was conducted in the absence of any commercial or financial relationships that could be construed as a potential conflict of interest. The reviewer RLC and handling Editor declared their shared affiliation, and the handling Editor states that the process met the standards of a fair and objective review.
