# Supplementary material for: NO-sGC Pathway Modulates Ca2+ Release and Muscle Contraction in Zebrafish Skeletal Muscle
Source: Front Physiol. 2017 Aug 23;8:607. doi: 10.3389/fphys.2017.00607 (PMC5572320; doi:10.3389/fphys.2017.00607)
Supplement: Supplementary file 1 [file Table1.pdf]

frontiers

Supplementary Material

NO-sGC pathway modulates Ca^{2+} release and muscle contraction in zebrafish skeletal muscle

Zhou Xiyuan[1], Rainer HA Fink[1] and Matias Mosqueira[1*].

1. Medical Biophysics Unit, Institute of Physiology and Pathophysiology, Heidelberg University Hospital, 69120 Heidelberg, Germany.
2. Department of Traumatic Surgery, TongJi Hospital affiliated to TongJi Medical College, Huazhong University of Science and Technology, NO 1095, Jiefang Road, Qiaokou District, Wuhan, China, Zip Code 430030

*** Correspondence:**
Matias Mosqueira
matias@physiologie.uni-heidelberg.de

Table SI. Summary of Ca^{2+}-transient biophysical parameters on Ca^{2+} release analyses obtained from electrically stimulated and isolated myocyte skeletal muscle of zebrafish larvae. Values expressed as Median; 25% - 75%. * p< 0.05; ** p< 0.01, *** p< 0.001 vs verapamil or ryanodine.

Biophysical Parameter	Peak (nM)	Time to Peak (ms)	Slope of Release (µM/s)
Verapamil (20 µM)	101.8; 82.69 – 146.1	3.80; 3.34 – 4.45	323.6; 243.7 – 413.2
SNAP + Verapamil	89.5; 76.4 – 102.8 *	4.90; 4.32 – 5.83	233.2; 205.7 – 295.1*
L-NAME + Verapamil	123.5; 91.3 – 140.1	3.88; 3.46 – 4.23	359.2; 307.2 – 422.6
Ryanodine (100 nM)	167.1; 133.1 – 246.4	5.57; 4.93 – 8.11	423.6; 362.2 – 620.2
SNAP + Ryanodine	139.9; 108.5 – 171.7	6.47; 4.11 – 8.01	387.9; 311.1 – 495.6
L-NAME + Ryanodine	172.2; 134.6 – 248.2	4.13; 3.69 – 5.05**	463.1; 406.5 – 495.6
Caffeine (250 µM)	603.8; 399.4 – 825.1	5.23; 4.26 – 7.00	863.5; 734.2 – 984.1
SNAP + Caffeine	349.6; 201.1 – 590.7	5.60; 4.63 – 6.80	687.5; 505.5 – 920.3
L-NAME + Caffeine	508.5; 387.2 – 911.8	4.30; 3.47 – 6.80	821.2; 706.2 – 957.4
