# Supplementary material for: NO-sGC Pathway Modulates Ca2+ Release and Muscle Contraction in Zebrafish Skeletal Muscle
Source: Front Physiol. 2017 Aug 23;8:607. doi: 10.3389/fphys.2017.00607 (PMC5572320; doi:10.3389/fphys.2017.00607)
Supplement: Supplementary file 2 [file Table2.pdf]

Supplementary Material

NO-sGC pathway modulates Ca^{2+} release and muscle contraction in zebrafish skeletal muscle

Zhou Xiyuan[1], Rainer HA Fink[1] and Matias Mosqueira[1*].

1. Medical Biophysics Unit, Institute of Physiology and Pathophysiology, Heidelberg University Hospital, 69120 Heidelberg, Germany.
2. Department of Traumatic Surgery, TongJi Hospital affiliated to TongJi Medical College, Huazhong University of Science and Technology, NO 1095, Jiefang Road, Qiaokou District, Wuhan, China, Zip Code 430030

*** Correspondence:**
Matias Mosqueira
matias@physiologie.uni-heidelberg.de

Table SII. Summary of Ca^{2+}-transient biophysical parameters on Ca^{2+} uptake analyses obtained from electrically stimulated and isolated myocyte skeletal muscle of zebrafish larvae. Values expressed as Median; 25% - 75%. * $p < 0.05$; ** $p < 0.01$, vs CPA or $NiCl_2$.

Biophysical Parameter	Peak (nM)	Uptake Time (ms)	Tau (s)
CPA (2.5 µM)	107.1; 54.2 – 141.4	5305; 4505 - 6067	81.41; 24.64 – 101.6
CPA + SNAP	100.6; 68.5 – 154.1	3648; 2419 - 7409	54.2; 29.8 – 129.1
CPA + L-NAME	119; 74.9 – 174.1	4255; 2766 - 5719	66.4; 37.9 – 101.5
$NiCl_2$ (2 mM)	83.2; 65.5 – 132.6	240.8; 99.1 – 511.8	7.60; 7.01 – 8.56
SNAP + $NiCl_2$	83.9; 65.5 – 99.5	139.0; 106.1 – 250.8	8.23; 7.57 – 9.03
L-NAME + $NiCl_2$	132.7; 95.3 – 167.5***	732.9; 194.5 – 1022*	7.91; 7.32 - 8.99
