# Supplementary figures and images for: NO-sGC Pathway Modulates Ca2+ Release and Muscle Contraction in Zebrafish Skeletal Muscle
Source: Front Physiol. 2017 Aug 23;8:607. doi: 10.3389/fphys.2017.00607 (PMC5572320; doi:10.3389/fphys.2017.00607)

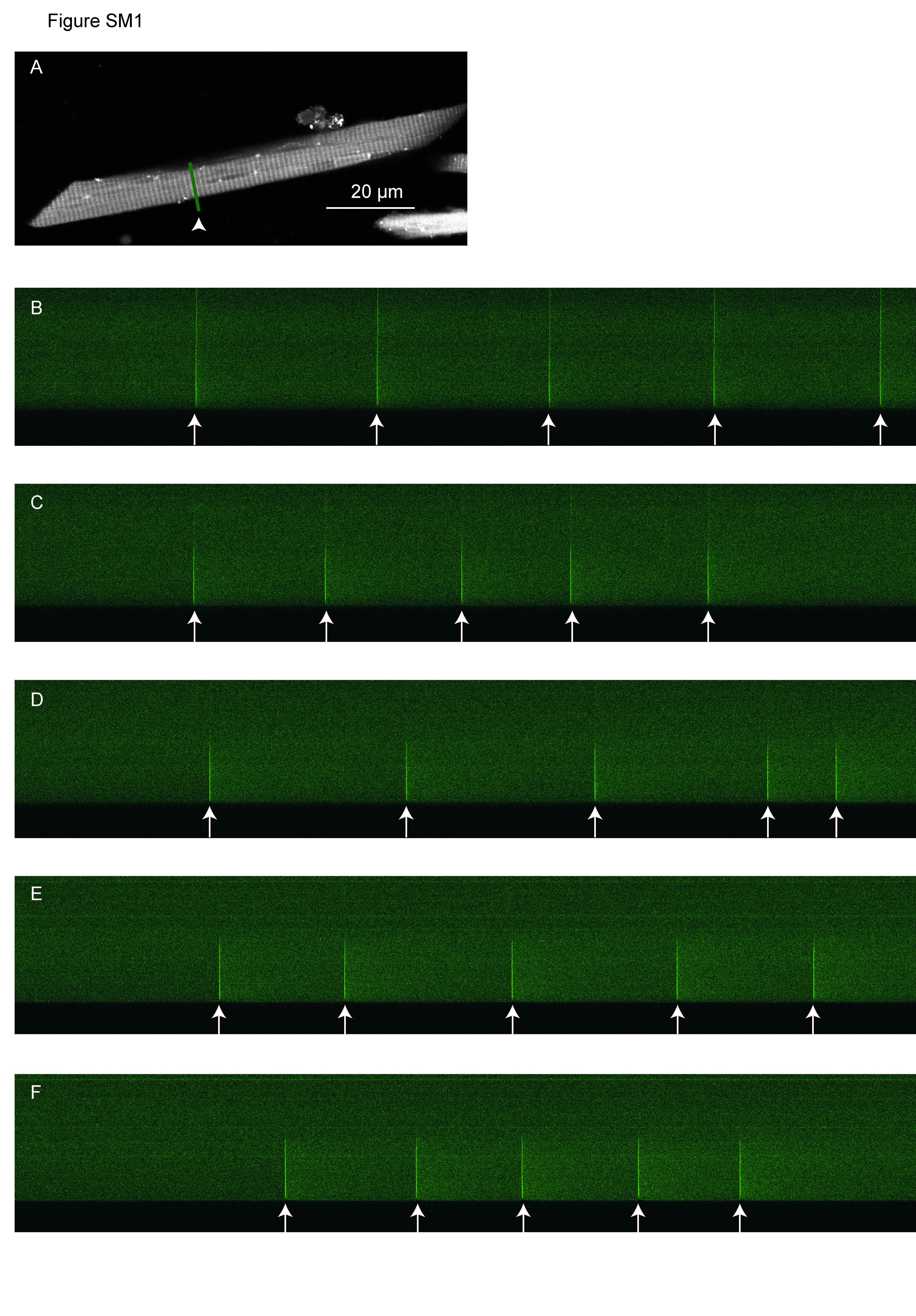

Supplement: Supplementary file 3 [file Image1.JPEG]
