# Supplementary material for: NO-sGC Pathway Modulates Ca2+ Release and Muscle Contraction in Zebrafish Skeletal Muscle
Source: Front Physiol. 2017 Aug 23;8:607. doi: 10.3389/fphys.2017.00607 (PMC5572320; doi:10.3389/fphys.2017.00607)
Supplement: Supplementary file 4 [file Image2.PDF]

Supplementary Material

NO-sGC pathway modulates Ca^{2+} release and muscle contraction in zebrafish skeletal muscle

Zhou Xiyuan[1], Rainer HA Fink[1] and Matias Mosqueira[1*].

1. Medical Biophysics Unit, Institute of Physiology and Pathophysiology, Heidelberg University Hospital, 69120 Heidelberg, Germany.
2. Department of Traumatic Surgery, TongJi Hospital affiliated to TongJi Medical College, Huazhong University of Science and Technology, NO 1095, Jiefang Road, Qiaokou District, Wuhan, China, Zip Code 430030

*** Correspondence:**
Matias Mosqueira
matias@physiologie.uni-heidelberg.de

1 Supplementary Figure

Figure SM1. Ca^{2+} transients do not occur in the entire isolated myocyte of 5-7 dpf zebrafish. A. Photomicrography of isolated myocyte of zebrafish larvae indicating with an arrowhead the position of the line-scanning. B-F. Line-scanning from the sub-sarcolemmal level of one side of the myocyte until the other with one micrometer step. In each level, several electrical stimulation was applied as indicated by the white arrows.
